# Does a Caesarean Section Scar Affect Placental Volume, Vascularity and Localization?

**DOI:** 10.3390/diagnostics12112674

**Published:** 2022-11-03

**Authors:** Diana Bokučava, Anda Ķīvīte-Urtāne, Pavels Domaševs, Laura Lūse, Natālija Vedmedovska, Gilbert G. G. Donders

**Affiliations:** 1 Department of Obstetrics and Gynecology, Rīga Stradiņš University, LV-1007 Rīga, Latvia; 2 Rīga Maternity Hospital, LV-1013 Rīga, Latvia; 3 Institute of Public Health, Rīga Stradiņš University, LV-1007 Rīga, Latvia; 4 Department of Public Health and Epidemiology, Rīga Stradiņš University, LV-1010 Rīga, Latvia; 5 Department of Obstetics and Gynecology, Antwerp University Hospital, 2650 Antwerp, Belgium; 6 Femicare VZW, Clinical Research for Women, 3300 Tienen, Belgium

**Keywords:** Caesarean section scar, three-dimensional ultrasonography, placental volume, placental vascular indexes

## Abstract

Caesarean section is associated with an increased risk of abnormal placental implantation and adverse pregnancy outcomes in subsequent pregnancies. Besides the placenta accrete spectrum, only a few of the previous studies focused on other placental development alterations in the scarred uterus. We assessed placental development deviations in the uterus with a Caesarean section scar by evaluating placental volume (PV) and vascular flow indexes. From 1 January 2021 until 31 March 2022, placental volumes and vascularization indexes (VI, FI, VFI) were prospectively measured by 3D power Doppler and VOCAL techniques in 221 patients attending the first trimester screening program. We also calculated the placental quotient to standardize PV to the gestational age. No statistically significant differences in the values of placental volume, placental quotient and placental vascularization indexes were detected between women with previous Caesarean section delivery or women with vaginal delivery. FI was significantly lower in nulliparous in the first trimester. The results of our study suggest that 3D placental evaluation was not able to detect placental development alteration in the uterus with a Caesarean section scar. Future research needs to verify whether 3D power Doppler and Vocal techniques can provide more information if used in an earlier gestational age.

## 1. Introduction

During at least three decades, there has been a problematic increase in the rate of the Caesarean sections (CS). The mean worldwide CS rate increased from 6.7% in 1990 to 21% in 2021 and may reach 30% by 2030 [1]. Amongst all long-term complications of CS, the most dangerous are the scar ruptures in future pregnancies and placenta accreta spectrum disease (PAS). It has been postulated that the CS increases the risk of PAS because of the damage of the endometrial basal layer and the hypoxic environment created by scar tissues [2], leading to abnormal placentation. Apart from this research on abnormal placentation in PAS, only a few studies have focused on other obstetric and perinatal outcomes in pregnancies after a previous CS delivery. Smith et al. [3] have reported an increased risk for unexplained stillbirth in pregnancies subsequent to the CS, compared to vaginal delivery. Kennare et al. [4] have found an association between the previous CS delivery and an increased risk of unexplained stillbirth, preterm birth, small for gestational age (SGA) and low-birth-weight infants.

Apart from gestational complications, there is evidence that a previous CS reduces the probability of obtaining a subsequent pregnancy by 10% [5]. Of 1317 women with a CS scar undergoing infertility treatment, only 15.9% ended in a live birth compared to 23.3% after a prior vaginal delivery [6].

This increased risk of such adverse perinatal outcomes and impaired fertility are scarring and deficient endometrium due to a damaged myometrium [7]. A destruction of the basal layer and spongiosus decidual layer may compromise implantation and contribute to an abnormal placentation and placental bed disruption [8]. Other hypotheses are the development of a postoperative niche in the area of the scar and subsequent intracavitary fluid accumulation, as well as altered endometrium immunology and increased endometrial inflammation due to the reduced recruitment of CD45+ cells during the endometrium secretory phase in a scarred uterus [9,10].

There is insufficient information on the impact of the scar tissue on impaired trophoblastic invasion and placentation, both main factors in the pathophysiology of the most common pregnancy complications [11,12,13]. Nowadays, the most readily available and informative modality for the assessment of placental development is ultrasound. It has proven itself as a crucial tool for differential diagnosis of abnormal placentations, providing an opportunity for decreasing pre- and intraoperative complications by timing early elective CS and an appropriate operation technique [14]. Furthermore, the studies evaluating the role of the placenta in pregnancy complications, such as fetal growth restriction (FGR) and preeclampsia (PE), have used advanced ultrasound technology to analyse placenta volume (PV) and placental vascularization, employing power Doppler parameters as vascular (VI), flow (FI) and vascularization flow indexes (VFI). VI provides information on how many vessels can be detected within the placenta and FI rather reflects the intensity of the placental blood flow [15,16]. In trying to combine the information on vascularity and the amount of transported blood cells, VFI was developed [15,17].

In the present study, we applied 3D ultrasound to assess placental development in the presence of a uterine scar. The aim is to evaluate the influence of a CS scar on placentation by analysing placental volume, VI, FI and VFI in the first and second trimesters of pregnancy.

## 2. Materials and Methods

**Figure 1:**
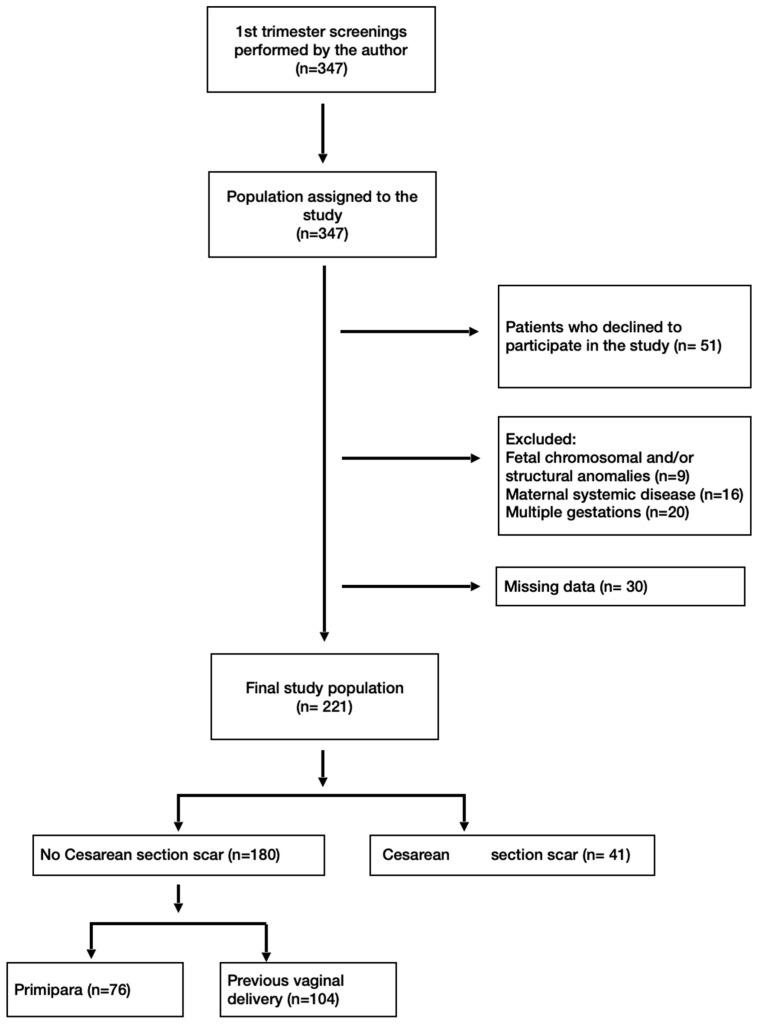
Flow chart illustrating patient selection and recruitment.

This prospective cohort study was undertaken at the Prenatal Diagnostic clinic of the Riga Maternity hospital from 1 January 2021 until 31 March 2022. After written informed consent, 347 consecutive, unselected women with singleton pregnancies presenting for the first trimester screening ultrasound between 11^+0^ and 13^+6^ weeks were invited to participate in the study (Figure 1). The patients were asked to fill out a health questionnaire regarding their previous obstetric history, the number of previous Caesarean deliveries and other medical data, such as age, BMI, smoking, chronic medical conditions and current pregnancy symptoms. The exclusion criteria were: multiple pregnancies, pregnancies with fetal morphological or chromosomal anomalies and severe maternal systemic disease. All the scans were carried out by sonographers with extensive experience in 3D ultrasound. The study was approved by the Ethics Committee of the Rīga Stradiņš University (NR. 17/26.10.2017).

All the ultrasound examinations were performed by abdominal ultrasonography (Volusone E8 Expert; GE Medical System, Chicago, IL, USA, convex 2–8 MHz probe). An initial 2D sonographic assessment provided the data about placental localisation and sonographic appearance, such as the presence of placental lacunes, ‘lakes’ or other visual alterations. Placental locations were recorded using the following subgroups: anterior, posterior and fundal. Gestational age (GA) was initially determined by measuring the crown-lump length (CRL) during the first trimester. During the second trimester, biparietal diameter (BPD), abdominal circumference (AC) and femur length (FL) were measured. Following the initial 2D sonographic examination, the power Doppler ultrasound was applied to image the placental vasculature. To obtain placental volume (PV) images and vascularization indices, we used the previously described methods [18,19]. The 3D PV was acquired by using a 3D trans-abdominal probe, holding it perpendicular to the placental plate, expanding the power Doppler colour and volume box size to include the entire placental area. All the power Doppler settings were standardized; the sweep angle was set to 85°. All the recordings were obtained by the author (D.B.) and stored for later analysis. After the first trimester screening, the same procedure was repeated during the second trimester screening. Measurements during the third trimester were not performed, as it is known from previous studies that PV measurements are not accurate due to the large placental surface that does not fit in the volume box [20].

In 50 randomly selected cases, the placental volume was measured in duplicate by a reference sonographer in order to calculate the intra-observer agreement.

**Figure 2:**
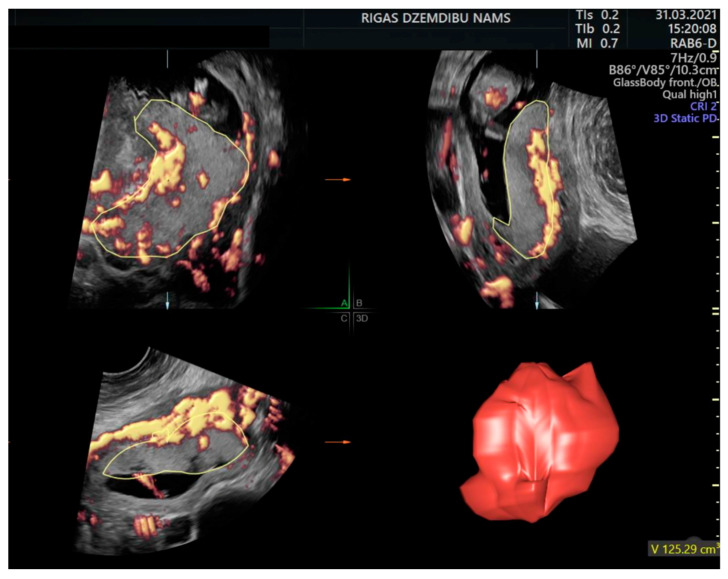
Assessment of the placental volume in the first trimester by the rotational technique using contour mode virtual organ computer-aided analysis.

The virtual organ computer-aided analysis (VOCAL) technique was used to analyse all the stored volumes off-line by one operator (DB). The contour mode in the VOCAL program was set to manual with 30° rotation steps. The software automatically calculated the placental volume after 6 contours of the placenta that were manually marked at the boundary (Figure 2). The VOCAL software also calculated vascular indices (VI, FI and VFI). To adjust for the placental volume increasing with gestational age, a placental quotient (PQ) (PV (cm^3^)/CRL (cm)) was calculated.

All the statistical analyses were performed using SPSS statistical software version 26.0. The Kolmogorov-Smirnov test was performed to assess the distribution of continuous variables. The Mann–Whitney U test was used to assess the differences in median values of the studied variables between the groups. A related-samples Wilcoxon signed rank test was used to assess the changes in the median or mean values of the studied variables between the pregnancy trimesters. A chi-square or Fisher exact test was used for the comparison of categorical variables between the study groups. The *p* < 0.05 value was considered statistically significant.

## 3. Results

As 51 patients declined to participate, the response rate to participate in the study was 86%. Nine patients were excluded because of fetal structural and/or chromosomal anomalies; 20 because of multiple gestation; and 16 women because of the presence of systemic disease (pregestational diabetes, chronic hypertension, antiphospholipid syndrome, lupus erythematosus, psoriatic arthritis). Further, 30 women failed to provide crucial information, despite more than one email sent to them with the request to clarify missing information. After the exclusion of these women, a final study group of 221 women was examined. The 41 women (18.6%) with a history of Caesarean delivery was called the ‘study group’, compared to 76 (34.4%) who had experienced at least one previous vaginal delivery (the control group). A third group consisted of 104 (47.1%) primipara patients (Figure 1).

The analysis of the demographic variables, such as age, BMI and smoking status, showed no differences between women with a previous Caesarean section and vaginal birth (Table 1). Primipara women were younger than the multiparous women; however, they showed no further demographic differences (Table 1).

The frequency of different placental locations between the groups in the first trimester is illustrated in Table 1. There was no difference in the placental localisation between the women with a previous CS or vaginal delivery (*p* = 0.38) and between nulliparas or the women with a previous vaginal delivery (*p* = 0.8).

There were no differences in PV between the women with a previous CS or vaginal delivery in the first and second trimester. The median PV in the first trimester in the CS scar group was 76.2 cm^3^, compared to 78.8 cm^3^ in the women with a previous vaginal delivery (*p* = 0.5), while this was 223.0 and 229.8 cm^3^, respectively, in the second trimester (*p* = 0.5) (see Figure 2). There were also no differences in the PQ values in the women with a previous CS or vaginal delivery (*p* = 1.0) (Table 2). In addition, PV and PQ did not differ between nulliparas and women with a previous vaginal delivery (Table 2).

**Figure 3:**
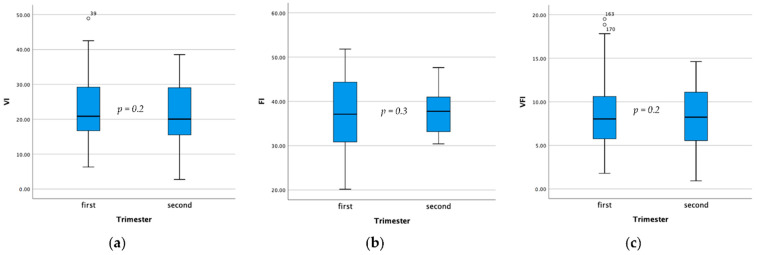
Box plots and whiskers plots demonstrating vascular index values in the first and second trimester in the women with a previous Caesarean section delivery. (**a**) Vascular index (VI), (**b**) flow index (FI) and (**c**) vascular flow index (VFI).

The indicators of the placental vasculature (VI, mean FI and mean VFI) are presented in Table 2. There were no significant differences in the vascular index values between women with a previous CS or vaginal delivery; neither in the first, nor in the second trimester of pregnancy. In the first trimester, however, FI was significantly lower in nulliparas than in the women with a previous vaginal delivery (*p* = 0.01). Finally, no differences were found in the VI, FI and VFI values between women with a past CS versus vaginal delivery, both during the first and second trimester (Figure 3). The summary of the main results is presented in Table 3.

## 4. Discussion

To the best of our knowledge, this is the first prospective study to evaluate PV and placental vascularization indexes by 3D ultrasound technology in order to compare placental development in pregnant women with and without a CS scar. During the previous decade, 3D ultrasound technology was used to explain placental developmental alterations in perinatal medicine. Nikolaides et al., for instance, described a smaller PV in pregnancies at 11 and 13 + 6 gestational weeks affected by trisomy 13 and 18 [21]. As an increased risk for subfertility, preterm birth, SGA, low birth weight and unexplained stillbirth has been described in women with a CS scar [3,4,5], this could imply that uterine scars predispose for impaired placentation and, as a consequence, lower fertility rates and fetal complications later in gestation.

The calculated first trimester PV values in women with and without a CS scar that we report in this study are in agreement with previous research [22,23], supporting evidence that the estimation of PV by the VOCAL technique in the first trimester is a quite accurate and reproducible technique. The second trimester PV, on the other hand, was slightly higher in our series than in the mentioned published studies [22,23]. This may be explained by the poorer inter-examiner reliability of the 3D PV measurements in the second trimester, as compared to the first trimester [24,25]. Indeed, after 20 weeks, the placental size is often larger than can be captured on an entire 3D sweep.

In order to explain the role of PV in the prediction of later pregnancy complications, Hafner et al. [26] demonstrated that PV measurements in the first and second trimester show different placental growth patterns, linked to different complications later in pregnancy. So, they demonstrated that a small PV at 12 weeks resulted more often in FGR; however, this intergroup PV difference disappeared by 12 weeks of pregnancy [26]. In pregnancies complicated by PE, PV had been larger than normal during the first trimester, but not between 16 and 22 weeks, when any difference in PV was no longer detected [26]. Finally, in pregnancies complicated with both PE and FGR, not only was PV smaller at 12 weeks, but subsequent placental growth also decreased significantly as compared to normal pregnancies [26]. Despite these encouraging results of one study, it is acknowledged that the findings of several researches on the prognostic meaning of PV have produced inconsistent and, at times, contradictory results [24,27,28,29]. In the present study, we did not find statistically significant differences between PV in the uterus with or without CS scars, either in the first or in the second trimester of pregnancy. PV also did not differ between nulliparas and women with a previous vaginal delivery.

As other authors suggested [23], we also took into consideration the fact that placental volume depends on the gestational age by using the placental quotient (PQ). Schwartz et al. [30] reported that a PQ below 1 can predict FGR with 56% sensitivity and 75% specificity. Using PQ measurements, Rizzo et al. were able to predict 67% of early PE (requiring delivery before 32 weeks) [17]. In the present study, there were no significant differences in the PQ values between groups; however, the study was not designed to measure different pregnancy outcomes, such as PE or FRG.

The fact that we did not find a different PV and PQ in the scar group in both trimesters seems to indicate that placental growth in the uterus with the CS scar is similar to the placental growth in the unscarred uterus. Therefore, to obtain further details on placental function, we also analysed placental vascular indexes in the first and the second trimesters. Previously, Pomorski et al. have demonstrated that in a normal pregnancy, placental vasculature grows in close relation to the increase in PV [23]. They noted that placental vascular indices (VI, FI, VFI) stayed constant through the pregnancy, even though PV increases with gestational age. Of importance, there may be an association between an abnormal shift in placental vascular indexes and pregnancy complications. Gonzalez–Gonzalez et al. have reported decreased VI as an independent predictor of FGR [31], though this result was contradicted by others’ findings [32]. Our data, showing similar VI values in women with and without a scar as well as in nulliparous women, seems to indicate that scar tissue does not interfere with the number or function of the placental vessels. Indeed, FI, reflecting placental blood flow intensity, was also reported to be reduced in the first and the second trimesters in FGR-complicated pregnancies [33]. In this study, FI was significantly lower in nulliparas in the first trimester, but not in the second trimester, and was not different in scarred versus intact uteri. This finding supports the concept that placental vascular development differs between nulliparas and multiparas and is in agreement with previous research [34]. Finally, VFI, reflecting the number of vessels in the placenta combined with the quantity of transported blood cells through it, has also been reported to be lower in PE and FGR cases [31,33,35]. Again, in our study, VFI values did not differ between the studied groups. Moreover, the constant VI, FI and VFI in the first and second trimesters in the CS group confirms that the development of placental vasculature is not influenced by a scar in the uterus, implying that other factors must be called in to explain the increased risk of adverse pregnancy outcomes after a CS in a previous pregnancy. Therefore, these results suggest that a 3D placental evaluation in the late-first and the second trimester is not able to detect placental development alterations in the uterus with a Caesarean section scar.

As our 3D placental investigations were only performed after 12 weeks of gestation, this could also potentially help to explain the failure to demonstrate a shift in placental placental developmental in the CS group. Indeed, comparing placental development, Ballering et al. found a different pattern of FI increase in nulli- versus multiparous women between 8 and 12 weeks of pregnancy; however, not after 12 weeks [34]. Therefore, in further studies, it would be interesting to evaluate placental development during earlier stages of gestation. In addition, including a higher number of pregnant women with the CS scar in their history and taking into account the time lapse since the CS took place, could lead to a better insight in the pathogenesis of placentation after CS scarring.

Therefore, in further studies, it would be interesting to evaluate placental development during the earlier stages of gestation. Furthermore, including in the research a higher number of pregnant women with the CS scar in their history and taking into account the time lapse since the CS took place could lead to a better insight in the pathogenesis of placentation after CS scarring.

## Figures and Tables

**Table 1:** Characteristics of the study groups and placental localisation distribution in the study groups. Abbreviations: CS, Caesarean section; SD, standard deviation; IQR, interquartile range; BMI, body mass index.

Variable		Previous CS		*p* *	Previous Vaginal Birth		*p* **	Nulliparas		Total	
		Mean (SD)	Median (IQR)		Mean (SD)	Median (IQR)		Mean (SD)	Median (IQR)	Mean (SD)	Median (IQR)
Age		34.8 (4.7)	36.0 (31.0–38.0)	0.06	33.0 (5.1)	33.0 (30.0–37.0)	<0.001	29.8 (5.1)	29.0 (26.0–33.0)	32.1 (5.4)	32.0 (28.0–36.0)
BMI		24.5 (4.4)	23.8 (21.8–26.7)	0.29	24.0 (5.1)	22.8 (20.3–26.7)	0.09	22.6 (3.7)	22.1 (19.9–24.3)	23.6 (4.5)	22.5 (20.3–25.9)
Smoking status during the pregnancy, n, %		2	5.7	0.60	3	2.9	0.70	3	3.9	8	3.7
Smoking status before the pregnancy, n, %		2	5.7	0.68	6	5.8	0.38	7	9.2	15	7.0
Placental localisation 1st trimester	Anterior	11	29.7	0.38	40	42.1	0.80	35	47.9	86	42.0
	Posterior	25	67.6		51	53.7		35	47.9	111	54.1
	Fundus	1	2.7		4	4.2		3	4.1	8	3.9

* *p* level between groups “previous CS” and “previous vaginal birth”; ** *p* level between groups “previous vaginal birth” and “nulliparas”. Data are given as numbers, percentages in parentheses.

**Table 2:** Distribution of vascular flow indexes in the first and second trimester in the study groups. Abbreviations: CS, Caesarean section; IQR, interquartile range; PV, placental volume; PQ, placental quotient; VI, vascular index; FI, flow index; VFI, vascular flow index.

Variable	Previous CS Median (IQR)	*p* *	Previous Vaginal Birth Median (IQR)	*p* **	Nulliparas Median (IQR)
PV 1st trimester	76.2 (52.8–100.8)	*p* = 0.53	78.8 (61.8–103.5)	*p* = 0.80	77.5 (54.5–102.2)
PV 2nd trimester	223.0 (178.4–343.7)	*p* = 0.55	229.8 (107.3–280.9)	*p* = 0.92	223.3 (177.2–309.7)
PQ	1.2 (0.7–1.8)	*p* = 0.99	1.20 (0.92–1.53)	*p* = 0.92	1.16 (0.7–1.5)
VI 1st trimester	20.8 (16.5–29.3)	*p* = 0.16	26.6 (17.8–33.2)	*p* = 0.21	22.8 (15.8–32.0)
VI 2nd trimester	20.0 (15.3–29.5)	*p* = 0.42	24.4 (14.5–33.3)	*p* = 0.14	14.9 (11.8–28.2)
FI 1st trimester	37.1 (30.7–44.3)	*p* = 0.62	38.8 (33.3–43.5)	*p* = 0.01	35.2 (30.3–38.7)
FI 2nd trimester	37.7 (32.5–42.9)	*p* = 0.38	40.3 (35.0–43.4)	*p* = 0.58	39.3 (34.3–41.3)
VFI 1st trimester	8.0 (5.7–10.7)	*p* = 0.20	9.3 (6.2–13.5)	*p* = 0.09	7.6 (5.2–10.5)
VFI 2nd trimester	8.2 (5.1–11.1)	*p* = 0.43	8.7 (5.8–11.6)	*p* = 0.12	5.7 (4.9–10.0)

* *p* level between groups “previous CS” and “previous vaginal birth”; ** *p* level between groups “previous vaginal birth” and “nulliparas”.

**Table 3:** The main results: the distribution of placental localisation, placental volume and vascular flow indexes. Abbreviations: CS, Caesarean section; IQR, interquartile range; PV, placental volume; PQ, placental quotient; VI, vascular index; FI, flow index; VFI, vascular flow index.

Variable		Previous CS N (%) or Median (IQR)	Previous Vaginal Birth N (%) or Median (IQR)	*p*
Placental localisation 1st trimester	Anterior Posterior Fundus	11 (29.7) 25 (67.6) 1 (2.7)	40 (42.1) 51 (53.7) 4 (4.2)	0.38
PV 1st trimester		76.2 (52.8–100.8)	78.8 (61.8–103.5)	*p* = 0.53
PV 2nd trimester		223.0 (178.4–343.7)	229.8 (107.3–280.9)	*p* = 0.55
PQ		1.2 (0.7–1.8)	1.20 (0.92–1.53)	*p* = 0.99
VI 1st trimester		20.8 (16.5–29.3)	26.6 (17.8–33.2)	*p* = 0.16
VI 2nd trimester		20.0 (15.3–29.5)	24.4 (14.5–33.3)	*p* = 0.42
FI 1st trimester		37.1 (30.7–44.3)	38.8 (33.3–43.5)	*p* = 0.62
FI 2nd trimester		37.7 (32.5–42.9)	40.3 (35.0–43.4)	*p* = 0.38
VFI 1st trimester		8.0 (5.7–10.7)	9.3 (6.2–13.5)	*p* = 0.20
VFI 2nd trimester		8.2 (5.1–11.1)	8.7 (5.8–11.6)	*p* = 0.43

## Data Availability

The data that support the findings of this study are available from the corresponding author upon reasonable request.
